# The stability and catalytic performance of K-modified molybdena supported on a titanate nanostructured catalyst in the oxidative dehydrogenation of propane

**DOI:** 10.1039/c8ra10598g

**Published:** 2019-04-16

**Authors:** Ehsan Goudarzi, Reza Asadi, Jafar Towfighi Darian, Amirhossein Shahbazi Kootenaei

**Affiliations:** Department of Chemical Engineering, Tarbiat Modares University P. O. Box 14115-143 Tehran Iran; Department of Chemical Engineering, Tarbiat Modares University P. O. Box 14115-143 Tehran Iran towfighi@modares.ac.ir +98 218 288 3311 +98 218 288 3311; Department of Chemical Engineering, Mahshahr Branch, Islamic Azad University Mahshahr Iran

## Abstract

Titanate nanotube supported molybdena was evaluated as a catalyst in the oxidative dehydrogenation of propane to propylene. The synthesized titanate nanotubes with high specific surface area were prepared by a hydrothermal method. The characterization of pristine nanotubes was performed *via* XRD, Raman, SEM, TEM and BET. The presence of hydrogen titanate nanostructure was confirmed in the bare support. Incipient wetness impregnation method was used to prepare MoTNT-*x* (*x* = 5, 10, and 15 wt% molybdena). The as-prepared catalysts' characterization was investigated using Raman, XRD, SEM, EDS, TEM, BET, TGA, and CHNS. Furthermore, H_2_-TPR was performed to explore reducibility of the catalysts. XRD and Raman results indicated development of the anatase phase in MoTNT-*x* catalysts upon calcination, along with specific surface area loss according to BET. Study of the catalytic performance of the samples showed an increase in catalytic activity and a significant drop in propylene selectivity with rising molybdena content. The maximum yield of propylene (about 9.3%) was obtained in 10 wt% of Mo content. The effect of potassium loading as a promoter in K/MoTNT-10 catalyst was also explored through characterization of the surface molybdena species and catalytic performance. Due to the presence of potassium, propylene yield increased from 9.3% to 11.3% at 500 °C. The stabilities of both catalysts were considered for 3000 min and showed only slight drops in propane conversion and propylene selectivity.

## Abstract 2

Titanate nanotube supported molybdena was evaluated as a catalyst in the oxidative dehydrogenation of propane to propylene.

## Graphical abstract

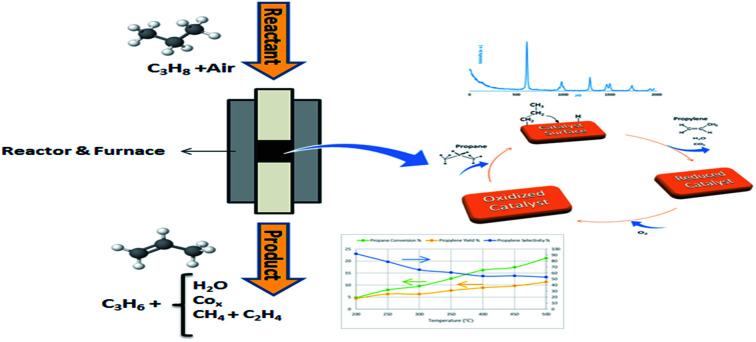

## 1. Introduction

The rapid development of human societies in the second half of the twentieth century was made possible by oil and gas, either as fuel or as a raw material. Unfortunately, fossil fuels do not contain olefins and mainly consist of saturated hydrocarbons and aromatics. Olefin production requires sophisticated technologies that are costly and require a large investment. Conversion of light alkanes to olefins has become one of the most interesting subjects for research over the last two decades.^1–4^ Olefins, because of their high reactivity, have a large role in producing polymers and other more valuable materials.

Propylene is a key product in the petrochemical industry, used as a feedstock to produce different polymers and intermediate products. Increasing demand for propylene in the global market, as well as general efforts to convert cheap and abundant raw materials and byproducts of petroleum refining processes into more valuable products, have resulted in substantial research into the oxidative dehydrogenation of propane.^5,6^ Catalytic dehydrogenation of alkanes is an endothermic reaction which requires a comparatively high temperature to achieve high yield. However, this high reaction temperature causes high thermal cracking, lowering alkane and coke formation and resulting in a drop in product yield and quick catalyst deactivation.^7^ Oxidative dehydrogenation of propane is a viable alternative to the catalytic dehydrogenation process with several benefits, such as being exothermic without any thermodynamic limitations. However, this approach suffers from problematic over-oxidation (combustion), which can decrease propylene productivity.^8^ A suitable catalyst for the ODH^1^ of propane must be able to effectively activate the C–H bond of propane and hamper unfavorable deep oxidation of propene to CO_*x*_.^9^ Transition metal oxides are the most important catalysts used in oxidative dehydrogenation of propane.^10–13^ The most extensively studied catalysts involve Mo/V/Ce based oxides.^14–19^ Much research has been done into molybdenum oxide catalysts supported on different metal oxides.^20–24^ Catalytic performance of the molybdena catalysts depends on the specific support, promoters, molybdena loading, calcination temperature, *etc.*^20,21,25^ It has been suggested that titania-supported molybdena catalysts are highly active in propane ODH,^21^ although conventional anatase titania suffers from low surface area.^10,21^

Recently, Kasuga^26^ presented a hydrothermal method to produce titanate nanotubes and TiO_2_ with large specific surface area and ion-exchange ability, appropriate for use as supports for active sites in catalysts. The procedure is uncomplicated, simple and cost-efficient; furthermore, it is an eco-friendly technique in comparison to the template method or anodic oxidation.^27^

In this article, we propose hydrothermally synthesized titanate nanotubes as a novel support, with a surprisingly high specific surface area, for K-doped molybdena catalyst to be used in oxidative dehydrogenation of propane *versus* conventional catalytic systems. Molybdena loading, potassium addition, calcination temperature and reaction temperature impacts were investigated through structure and catalytic performance of titania-supported K/Mo catalysts. Catalyst deactivation phenomenon was explored in the system to study stability of the K-promoted and non-promoted catalysts for 3000 min.

## 2. Experimental

### 2.1. Synthesis of titanate nanotube

Generally, to prepare the catalyst support, 1.7 g of Degussa TiO_2_ P25 was added to 150 ml of 10 M aqueous solution of NaOH (Merck) in an exothermic mixing process. The prepared mixture was stirred for 30 min, then transferred into a sealed Teflon-lined stainless-steel autoclave, filling about 80% of the volume. The sample was kept in an oven at 140 °C for 24 h, then the resulting mixture cooled at room temperature and was placed in a centrifuge for 15 min. The materials were washed using a weak acid solution of 0.1 M HNO_3_ until the pH of the rinsing solution attained about 1. The sediment was then rinsed with doubly deionized distillated water until the passing water reached pH 7. The obtained sample was dried at 110 °C for 12 h.

### 2.2. Catalyst preparation

Two types of catalysts were prepared by the incipient wetness impregnation method. The first type of catalyst (MoTNT-*x*, where *x* is the wt% of MoO_3_) involved a certain amount of MoO_3_ supported on titanate nanotubes. Briefly, a calculated amount of ammonium heptamolybdate was added to a measured volume of doubly deionized water that corresponded to the total pore volume of the support. Then, the support was added to the solution. The mixture was stirred at 70 °C until forming a paste. The resulting sample dried for 12 h at 110 °C to make a powder. The powder was calcined in static air for 3 h at 500 °C. After cooling to room temperature, the calcined sample underwent a forming process and 60–100 mesh size was chosen for the catalytic activity and deactivation tests.

In addition to MoO_3_, the second type of catalyst (MoK*y*TNT-*x*, where *x* and *y* are the wt% of MoO_3_ and the K : Mo molar ratio, respectively) involved a specific amount of KOH supported on titanate nanotubes. In the first step of catalyst preparation, a calculated amount of KOH was added to the measured ammonium heptamolybdate before stirring in deionized water. Next steps were identical to the preparation procedure for the first type of catalyst.

### 2.3. Catalyst characterization

X-ray diffraction (XRD) patterns of the catalysts and titanate nanotubes were recorded on a Philips PW1800 diffractometer using Cu Kα radiation (*λ* = 0.15418 nm). The intensities were determined for all of the synthesized samples with 2*θ* range from 5° to 70° at a step-size Δ(2*θ*) of 0.03° and a count time of 2 s per step. The indexing of attained spectra was carried out by comparison with JCPDS files (Joint Committee on Powder Diffraction Standards). The mean crystallite size of the sample was estimated by Scherrer's equation, from the XRD line-broadening measurement, as follows:1
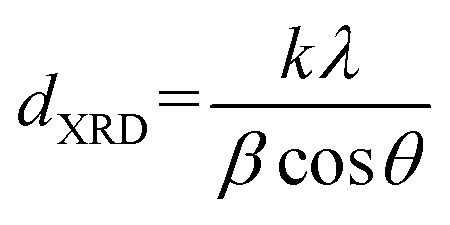
where *k* is a constant equal to 0.9 (shape factor), *λ* is the wavelength of the X-ray in nanometers, *θ* is the diffraction angle and *β* is the true half-peak width.

Raman spectra were recorded with a Bruker (model SENTERRA (2009)) spectrophotometer. A diode laser (*λ* = 785 nm) operating at 25 mW was employed as Raman excitation source with a germanium thermoelectrically cooled charged couple device (Andorf) as detector.

Specific surface areas of the catalysts were determined by N_2_ adsorption/desorption at −196.15 °C using BET method (BELSORP Mini II apparatus) with a ten point-isotherm. The samples were degassed for 2 h at 200 °C prior to nitrogen adsorption.

For transmission electron microscopy (TEM), the material was dispersed at room temperature in isopropanol and an aliquot of the prepared sample was deposited onto perforated carbon foil supported on a copper grid. The investigations were made on a Zeiss EM 900 microscope.

Scanning electron microscopy (SEM) was performed by a TESCAN MIRA3 Model apparatus equipped with an analytical system for energy dispersive X-ray spectrometry (EDS) to determine the morphology of the prepared samples.

The H_2_-temperature programmed reduction (H_2_-TPR) experiments were carried out in a Quantachrome CHEMBET-3000 apparatus using 20 mg of samples with 10 sccm of 7.0% H_2_ in air with concomitant temperature leveling up to 700 °C at a heating rate of 10 °C min^−1^. A thermal conductivity detector (TCD) monitored hydrogen consumption by analyzing the TPR reactor effluent. For quantitative purposes, the TCD signal was calibrated by reduction of Ag_2_O under similar conditions. Prior to this analysis, the sample was oxidized in flowing air at 200 °C for 1 h.

Thermal gravimetric analysis (TGA) was performed using a Netzsch-TGA 209 F1 thermo-gravimetric analyzer in air atmosphere from 25 to 900 °C with a heating rate of 10 °C min^−1^.

Elemental analysis was performed using Perkin Elmer 2400 Series II CHNS analyzer. The CHNS analysis was based on the classical Pregl-Dumas technique using a furnace temperature of 1100 °C.

### 2.4. Catalytic activity and deactivation study

**Fig. 1:**
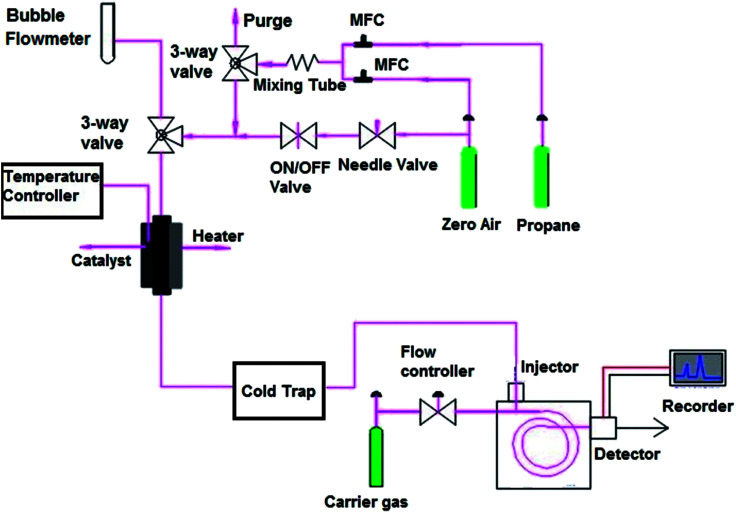
Schematic of the fixed-bed quartz reactor.

Activities of the catalysts in oxidative dehydrogenation of propane were investigated in a microflow fixed-bed quartz reactor (Fig. 1) with an internal diameter of 6 mm, external diameter of 7 mm and length of 50 cm at atmospheric pressure. An electric furnace was used to heat the reactor together with a thermocouple type K inside the catalyst bed. The catalytic bed was placed in the middle of the furnace (low thermal variations within a suitable longitudinal range). Blank runs were executed with the reactor packed with quartz wool at 500 °C to show the negligibility of propane conversion in the vacant reactor. 100 mg of catalyst with a mesh size of 60–100 diluted with 100 mg silicon carbide for better thermal distribution of the samples were utilized for each test. The samples were kept in the center of the reactor on a piece of quartz wool. The catalyst was pretreated using 20 sccm dry air flow at atmospheric pressure with heating rate of 10 °C min^−1^ up to 300 °C; it was then cooled under air flow to 200 °C. Next, a mixture of propane (99.8%) and air (99.995%) was fed into the catalytic reactor bed using calibrated mass flow rate controllers. The feed flow was mixed in a chamber with molar ratio of propane/O_2_ equal to 1 and flow rate of 100 sccm before contact with the catalyst. The temperature was increased in a stepwise fashion, with steps of 50 °C up to 500 °C, such that every step of the reaction lasted 30 min.

Analysis of the composition of the reaction's output products was performed on-line by a VARIAN CP-3800 gas chromatography device equipped with two flame ionization detectors (FID) and a methanizer (Ru/Al_2_O_3_). Carbon balance was established for all the catalytic tests, within 5%. Conversion, selectivity, and yield are all calculated based on carbon atoms as follows:2
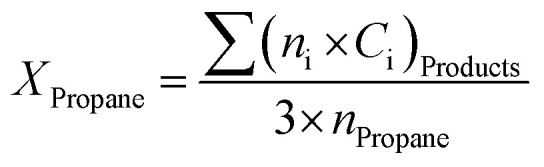
3
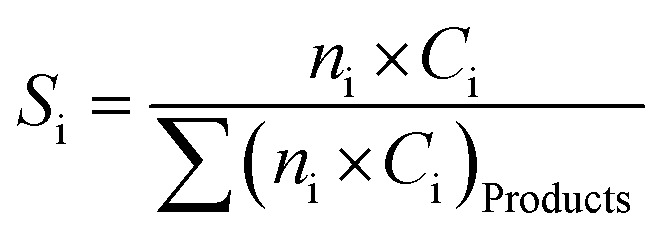
4
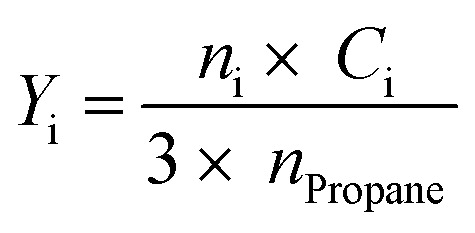
Here, *X*_Propane_ represents propane conversion. *S*_i_ and *Y*_i_ stand for product i selectivity and yield, respectively; *n*_i_ and *c*_i_ indicate the moles of molecule i and the number of carbon atoms in molecule i, respectively.

The turnover frequency (TOF) per molybdena atomic unit is determined according to eqn (5), with a diagram provided in Fig. 8d.5
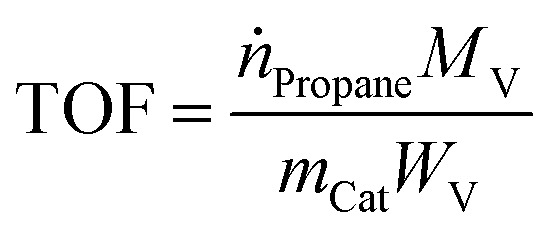


In this relation, *ṅ*_Propane_ is the molar flow rate of propane in the feed (1.17547 × 10^−5^ mol s^−1^), *X*_propane_ represents the propane conversion, *M*_V_ is the molecular weight of molybdena (95.96 g mol^−1^), *m*_Cat_ denotes the catalyst's mass (g), *W*_v_ is the weight fraction of molybdena in the catalyst, and TOF is the turnover frequency (s^−1^).

Deactivation studies of the catalysts were performed at 500 °C for 3000 min continuously under feed flow with similar conditions to the activity tests. The reactor's effluent was sampled with a time step of 150 min. Following completion of the deactivation study, the feed flow was changed to He to conserve the catalyst state for further studies. The catalyst was cooled to room temperature in the reactor.

## 3. Results and discussion

### 3.1. XRD analysis

**Fig. 2:**
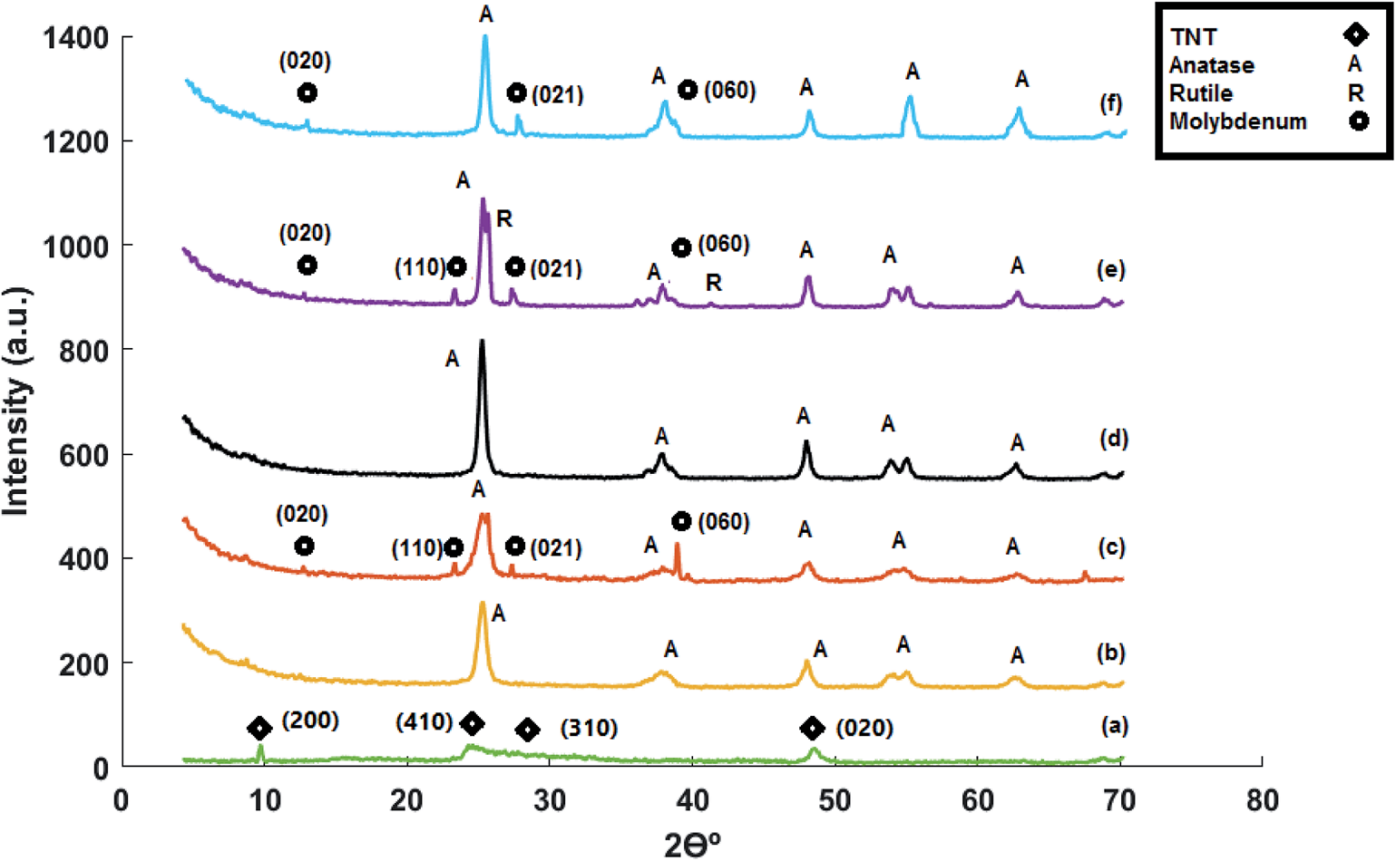
XRD patterns of (a) acid-treated titanate nanotubes, (b) MoTNT-5, (c) MoTNT-10, (d) MoK0.1TNT-10, (e) MoTNT-15, and (f) spent MoK0.1TNT-10.

XRD spectra related to the titanate nanotubes and developed catalysts are provided in Fig. 2. Understanding the crystalline structure of titanate nanotubes is usually difficult due to broad reflections in XRD pattern, ascribed to the small size of nanotubes.^10^ In addition, titanate nanotubes are relatively unstable and can experience various phase alterations during catalyst preparation methods such as acid washing and calcination, resulting in ambiguity of the exact crystal structure of titanate nanotubes.^28^

In Fig. 2a, the peaks at 10.7°, 24.2°, 28.6°, and 48.3° can be attributed to planes 200, 110, 310, and 020, respectively, in H_2_T_i5_O_11_·H_2_O (JCPDS: 44-0131). Accordingly, it seems that the hydrothermal process decomposes the structure of the precursor Degussa TiO_2_ P25 in the presence of alkaline solution, thereby forming a completely new structure.

The XRD pattern related to MoTNT-5 in Fig. 2b only includes the anatase phase (JCPDS: 21-1272). Absence of any MoO_3_ diffraction pattern might be a result of full dispersion of molybdena species on the support surface or formation of very tiny molybdena crystals on the surface, whose size is below the device detection capacity.^23,25,29^ The molecular structure of molybdena is such that, with increase in molybdena loading and following mono-molybdena species, we observe growth of two-dimensional and three-dimensional species of polymolibdate^30^ and a propensity for MoO_3_ crystals to develop, though these species can coexist as well. The probability of formation of polymeric molybdena species increases as a monolayer coating is approached.

A monolayer coating with a specific surface area of 41 m^2^ g^−1^ for commercial anatase support has been obtained in 3.9% loading of molybdena.^21^

The XRD pattern of MoTNT-10 catalyst represents the presence of molybdena oxide along with anatase phase (JCPDS: 35-0609), as the peaks shown in Fig. 2c can be attributed to planes 020, 110, 021, and 060, respectively. The relevant peaks have very low intensity, suggesting formation of few molybdena crystals in conjunction with mono- and poly-molybdate species. Peaks associated with rutile phase were not detectable.

Presence of potassium in the catalyst K0.1MoTNT-10 improved dispersion of molybdena species on the support surface. Further, peaks related to molybdena crystals were not detectable, suggesting formation of no molybdena crystals or only microcrystals of size smaller than the detection capacity of the device. Moreover, the intensity of the peak related to anatase phase increased in comparison to the catalyst MoTNT-10. It can be stated that the presence of potassium diminished the conversion of anatase phase into rutile. Superficial species of potassium were not detected, due to its low level in the sample.

The XRD pattern of MoTNT-15 catalyst suggests increased intensity of peaks related to molybdena crystals and diminished intensity of the anatase phase peaks compared to MoTNT-10 catalyst. It can be concluded that the number and size of MoO_3_ crystals increase with increasing molybdena loading on the titania support surface. Further, as shown in Fig. 2e, peaks associated with rutile phase (planes 110 and 111) were detectable in the XRD pattern of the catalyst with increase in molybdena loading (JCPDS:21-1276).

Disappearance of the index peak 2*θ* = 10.6°, related to the structure of titanate hydrogen nanotubes, refers to the molybdena loading and catalyst calcination resulting in alteration of the nanotube structure and its phase conversion to the anatase phase.

Using Scherrer's equation (eqn (1)), the average crystallite size of the catalysts was calculated considering the peak located at 2*θ* = 24.2° as the characteristic peak and the results are shown in Table 1. Crystallite size shows a growing trend with increasing Mo loading, which can be attributed to further formation of polymeric and crystalline molybdena species and destruction of the support structure because of anatase to rutile conversion. Lower crystallite size in MoK0.1TNT-10 can be ascribed to the presence of potassium in the support surface structure.

**Table 1:** Specific surface areas and H_2_-TPR results of prepared catalysts

Sample	BET surface area m^2^ g^−1^	H_2_-TPR results *T*_max_ (°C)	Mean crystallite size (nm)
Degussa TiO_2_ P25	49	—	—
Acid treated TNT	401	—	—
MoTNT-5	76	485.5	23.3
MoTNT-10	69	496.2	28.5
MoK0.1TNT-10	74	521.5	23.4
MoTNT-15	44	542.6	34.7
Spent MoTNT-10	56	—	—
Spent MoK0.1TNT-10	67	—	—

The XRD pattern of spent K0.1MoTNT-10 catalyst in Fig. 2f shows lower intensity of anatase characteristic peak at 2*θ* = 24.2° compared to the unspent one. Moreover, the presence of molybdena oxide in the catalyst is proved, as the peaks can be attributed to planes 020, 021, and 060, suggesting formation and growth of both molybdena crystals and its polymeric species. It should also be mentioned that no rutile phase was seen in the XRD pattern of the spent catalyst.

### 3.2. Raman analysis

**Fig. 3:**
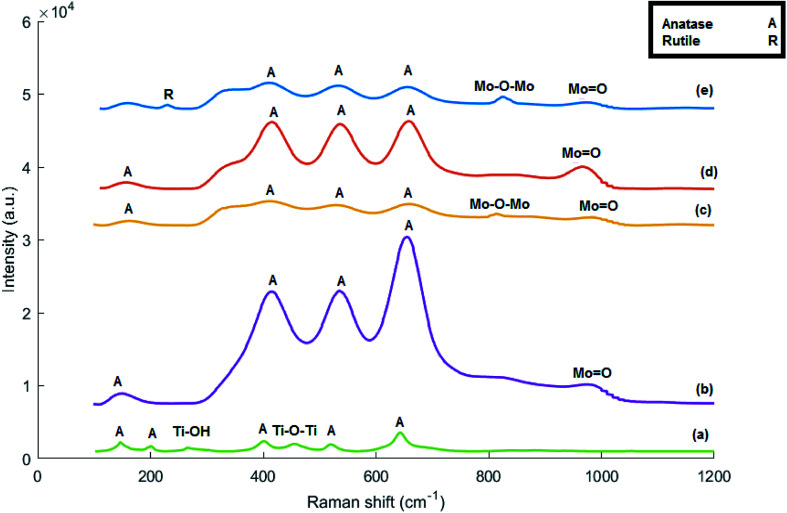
Raman spectra of (a) acid-treated titanate nanotubes, (b) MoTNT-5, (c) MoTNT-10, (d) MoK0.1TNT-10, and (e) MoTNT-15.

The Raman spectra of titanate nanotubes are provided in Fig. 3a. The shoulders at 145 cm^−1^ and 402 cm^−1^ can be attributed to the E_g_ mode of the anatase phase. The intensity of these bands increases during the acid washing stage with the decrease in sodium present in the titanate nanotube structure.^31^ This suggests the effect of sodium in prevention of conversion of titanate nanotubes to anatase. Considering the intensity of the 145 cm^−1^ and 402 cm^−1^ bands observed in the Raman spectrum of the synthesized sample, it can be stated that there is little sodium in the structure. The band at 266 cm^−1^ can be assigned to Ti–OH bonds, which are important for formation and stability of titanate nanotubes. By calcination of the nanotubes at high temperatures, two Ti–OH bonds are merged to form a Ti–O–Ti bond by releasing H_2_O. This is the cause of destruction of the tubular structure of the nanotube and formation of anatase phase.^10,32^

The band at 448 cm^−1^ is related to vibrations of the Ti–O–Ti bond and that at 903 cm^−1^ is associated with Na–O–Ti bond.^33^ This latter band did not exist in the Raman spectra of our synthesized support, suggesting absence or minimal existence of sodium in the structure of the synthesized titanate nanotube. The bands above 650 cm^−1^, including the bands at around 830 and 926 cm^−1^, are sensitive to humidity and, during the drying process of the support, they become weaker and move to higher frequencies. These bands can be assigned to superficial vibrational modes.^31^ The bands at 200, 510, and 635 cm^−1^ can be attributed to the anatase phase.^25,29,34,35^

The Raman spectra related to MOTNT-5 catalyst in Fig. 3b has bands at 410, 520, and 645 cm^−1^, the dominant bands of anatase. No band related to rutile phase was observed. Moreover, no peak suggesting the presence of crystal molybdena species was observed, in line with the obtained results from XRD. The band at 975 cm^−1^ is related to the Mo=O bond in monomer and polymer species of molybdena.^25,36^ The band at 265 cm^−1^ disappeared from the Raman spectra of MoTNT-*x* catalysts, suggesting the effect of molybdena loading in destruction of the titanate nanotube structure and its conversion to anatase phase. The reduction in the intensity of the bands associated with anatase phase with increase in the molybdena loading on the support surface is notable in Raman spectra of catalysts in Fig. 3c and d.

The broad and not very intense band at 820 cm^−1^ in the MoTNT-10 catalyst spectrum in Fig. 3c can be attributed to Mo–O–Mo bonds in the crystalline or polymeric structure of molybdena species.^37,38^ Comparison of the catalyst MoTNT-10 with MoTNT-5 indicated that the presence of a band at 820 cm^−1^ is followed by reduction in the intensity of the band related to mono-molybdena species and its transference to the higher frequency of 980 cm^−1^, which is in line with the results of other reports.^20,25^ This suggests reduction in the presence of monomer molybdena species on the support surface with the increase in its loading. This transition is due to the altered length of the Mo=O bond and can be attributed to decreased interaction with the support and production of three-dimensional polymer species of molybdena.^20,39^ This is fully compatible with the obtained results from XRD. No band related to rutile phase was observed.

Presence of potassium in the structure of K0.1MoTNT-10 catalyst, as shown in Fig. 3d, caused the disappearance of the 820 cm^−1^ band associated with crystal species of molybdena from its Raman spectra and improvement of dispersion of molybdena species on the support surface, such that the intensity of the band related to Mo=O bond increased significantly and transferred to the lower frequency of 966 cm^−1^.^29^ The intensity of bands related to anatase phase increased in response to presence of potassium compared to MoTNT-10 catalyst. No band associated with rutile phase was observed. Due to the low level of potassium in the catalyst, no band suggesting the presence of superficial species of potassium was observed. Watson *et al.*^29^ attributed the bands at 900–950 cm^−1^ to K_2_MoO_4_ and K_2_Mo_2_O_7_ species in catalysts with larger amounts of potassium.

The Raman spectrum of MoTNT-15 catalyst (Fig. 3e) showed increased intensity of the bands related to molybdena crystals in comparison to MoTNT-10 catalyst. It can be concluded that the number and/or the size of MoO_3_ crystals increases with elevation of molybdena loading on the titania support surface. Eventually, by developing a bulk phase on the surface, it causes diminished access to the active sites of the catalyst.^20^ Further, a band associated with rutile phase was observed at 230 cm^−1^, in line with XRD results.

### 3.3. The specific surface area of BET

The BET surface area of the acid-washed nanotubes was found using a BET isotherm. It is remarkable that the nanotubes reached a specific surface area of 401 m^2^ g^−1^, while the area of TiO_2_ P25, precursor of the nanotubes, was calculated to be 49 m^2^ g^−1^, as shown in Table 1. Hydrothermal method is successful in preparation of a support with a high specific surface area. The acid treating process causes increase of the surface area by a significant value, but the issue of thermal stability also comes into play.^40,41^ The specific surface area of acid washed nanotubes obtained through hydrothermal method has been reported at 404 m^2^ g^−1^, 408 m^2^ g^−1^ and 325 m^2^ g^−1^ in the literature.^10,42,43^

As can be observed in Table 1, the BET specific surface areas of prepared catalysts are lower than that of the synthesized nanotube, where the area diminishes with increase in molybdena loading. As the nanotubes have not been calcined, calcined Mo-catalysts showed a surface area much lower than that of the support. The specific surface areas of MoTNT-*x* with loading of 5, 10, and 15 wt% reach 76, 69, and 44 m^2^ g^−1^, respectively. It is also observed that the presence of potassium in K0.1MoTNT-10 catalyst causes the surface area to reach 74 m^2^ g^−1^, an enhancement compared to MOTNT-10 catalyst. This could be due to improved dispersion of molybdena species on the surface (increased level of monomeric species of molybdena), also observed in XRD and Raman analyses. The probability of pore plugging is considerable in response to polymer and crystal species of molybdena. Smaller species cause better dispersion and a higher specific surface area.^20^ Watson *et al.*^29^ reported elevation of the specific surface area of catalysts synthesized by hydrothermal method in response to the addition of potassium, which is in line with our observations. Further decline in specific surface area of MoTNT-15 could be due to higher level of molybdena so that it's more than monolayer coverage of the support surface,^21^ because further loading of molybdena causes weaker interaction between its species and the support surface. This causes formation of crystal species of molybdena, which are the cause of support pore blocking.^20,21^ Furthermore, this surface area reduction can be attributed to destruction of the nanotube structure, which includes stages such as thinning of the walls, blending of nanotubes, breakdown of nanotubes, and conversion to nanoparticles or nanorods.^44^

### 3.4. SEM, TEM and EDS

**Fig. 4:**
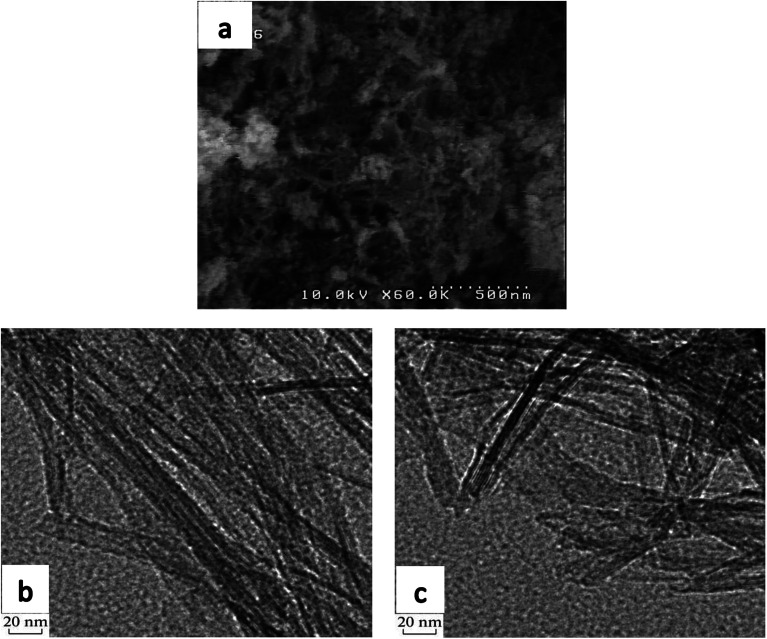
SEM (a) and TEM (b and c) images of titanate nanotubes.

SEM imaging was carried out to investigate the morphology of the synthesized support, shown in Fig. 4a, confirming the formation of nanotubes without discernible impurities.

TEM images of uncalcined TNT, provided in Fig. 4b and c, show a tubular morphology which proves titanate nanotube formation. Existence of this phase in the support structure has already been proven in XRD and Raman analyses. The prepared nanotubes have an external diameter of around 10 nm and lengths of 40–80 nm.

**Fig. 5:**
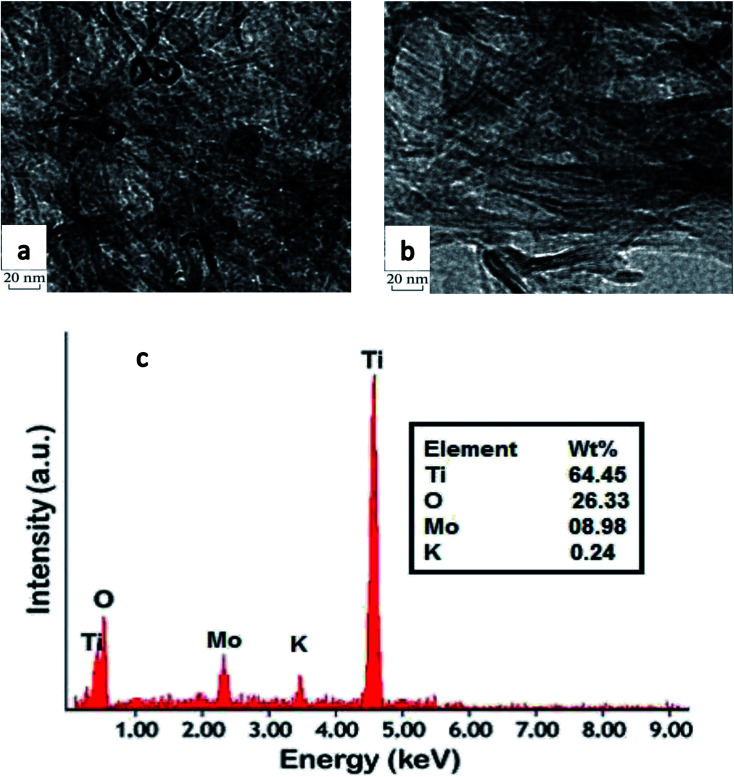
TEM images of (a) MoTNT-10 and (b) MoK0.1TNT-10; (c) EDS of MoK0.1TNT-10.

As shown in Fig. 5a, the tubular structure of the support changed completely after addition of 10 wt% molybdenum to TNT and calcination at 500 °C. In fact, some nanotubes broke into nanoparticles and a random mix of nanotubes and nanoparticles is observed. In contrast, the presence of potassium in MoK0.1TNT-10 catalyst increased the structural stability of TNT. The TEM image in Fig. 5b shows that the TNT tubular structure is fairly stable and fewer nanoparticles are formed.

EDS analysis, presented in Fig. 5c, proves the presence of potassium ions in the structure of the catalyst, strong evidence that the impregnation method successfully added potassium to the catalyst support structure.

### 3.5. H_2_-TPR analysis

**Fig. 6:**
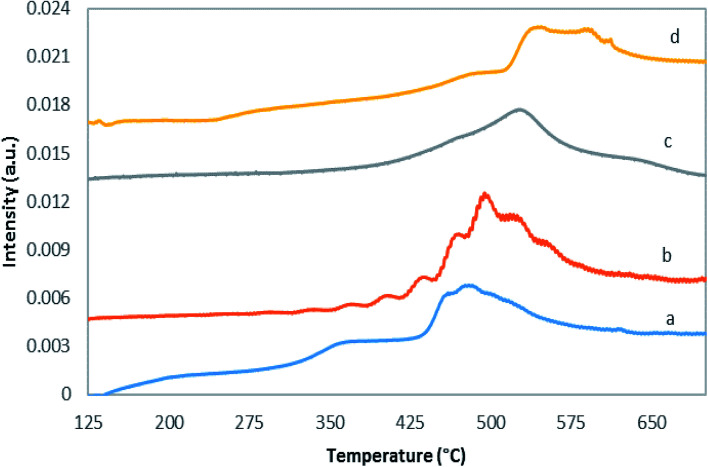
H_2_-TPR profiles of (a) MoTNT-5, (b) MoTNT-10, (c) MoK0.1TNT-10 and (d) MoTNT-15.

Temperature programmed reduction with hydrogen is a method to investigate the reducibility of catalysts, as presented in Fig. 6 and Table 1. Based on the literature, reduction of pure molybdena oxide involves several stages.^45,46^ The TPR results of bulk molybdena oxide show the presence of two peaks at 1040 and 1270 K, which were related to reduction of MoO_3_ to MoO_2_ (Mo^6+^ to Mo^4+^) and MoO_2_ to Mo. Compared to the bulk MoO_3_ TPR profile, TiO_2_-supported MoO_3_ displays a noticeable decrease in the maximum hydrogen consumption temperature.^47^ This enhancement suggests formation of more monomeric and polymeric molybdena species on the support surface in response to increased interaction with the surface. Note that monomeric, polymeric, and crystalline genera can coexist in MoO_*x*_/TiO_2_ catalysts.

As can be observed in Fig. 6 and Table 1, with increase in the molybdena loading, the maximum hydrogen consumption temperature increases from around 485 °C to 542 °C, which is consistent with polymeric and crystalline MoO_*x*_ presence. It is difficult to precisely distinguish MoO_*x*_ species peaks in H_2_-TPR profiles, because different species can reduce simultaneously.

The maximum reduction temperature for MoK0.1TNT-10 catalyst was 521.5 °C. It is noteworthy that this temperature is higher than the MoTNT-10 catalyst maximum reduction temperature, which can be attributed to presence of potassium within the sample structure. The increase of the reduction temperature can be attributed to reduction of K-affected monomeric species.^10^

### 3.6. TGA and CHNS of spent MoK0.1TNT-10

**Fig. 7:**
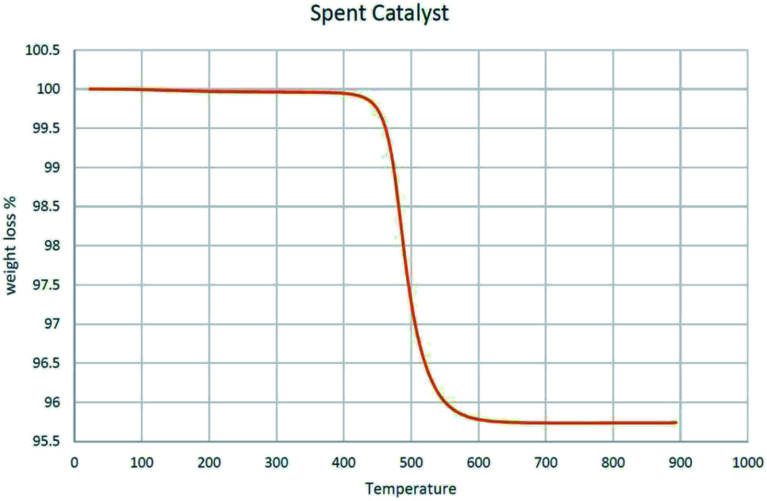
TGA of spent MoK0.1TNT-10 catalyst.

Spent MoK0.1TNT-10 was heated from 25 to 900 °C at a heating rate of 10 °C min^−1^ in a thermogravimetric analyzer to measure its mass over time. Fig. 7 shows that the sample had a negligible mass loss below 450 °C, which was expected, as it had previously participated in the ODH reaction at 500 °C for 3000 min. A mass loss of about 4% was observed in the range of 450 °C to 600 °C that is attributed to the burning of coke formed through the ODH reaction. The low amount of mass loss is because the nature of oxidative dehydrogenation of propane does not allow significant coke formation. No obvious weight loss was seen at temperatures above 600 °C, demonstrating high thermal stability of the spent catalyst.

CHNS analysis was performed to determine the elemental composition of spent MoK0.1TNT-10. The results, shown in Table 2, imply that the carbon content in the sample is less than 5%, which is compatible with the observation in the TGA analysis. Indeed, the higher the level of carbon in the catalyst, the higher the probability of surface pore blockage, which results in specific surface area reduction.

**Table 2:** CHNS of Spent MoK0.1TNT-10 catalyst

Catalyst	C%	H%
Spent MoK0.1TNT-10	4.02	0.65

The analyses prove that coke formation had a notable role in neither the pore plugging nor the reduction of active sites on the surface of the catalyst.

### 3.7. Catalytic test results

**Fig. 8:**
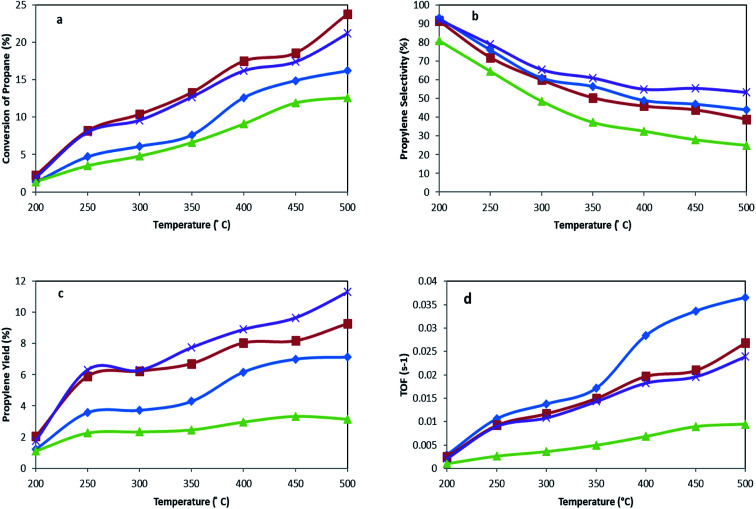
(a) Conversion of propane, (b) propylene selectivity, (c) propylene yield, and (d) TOF for MoTNT-5 (♦), MoTNT-10 (■), MoK0.1TNT-10 (×), and MoTNT-15 (▲).

**Fig. 9:**
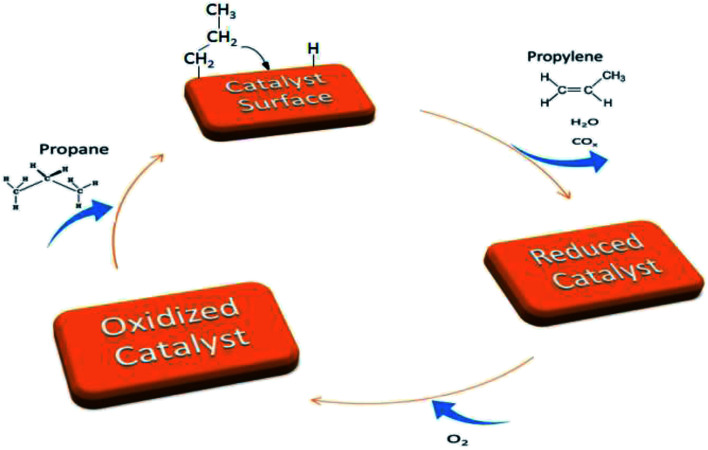
Mars–Van–Krevelen mechanism (oxidation–reduction).

The catalytic performance of titanate nanotubes and calcinated catalysts in the oxidative dehydrogenation of propane was examined within the thermal range of 200–500 °C, as shown in Fig. 8. In addition to propylene, other products, including H_2_O, CO, and CO_2_, were also produced. Increasing the reaction temperature to 500 °C, slight amounts of methane and ethylene were observed in the output products, but their selectivities remained below 0.2%. The oxidative dehydrogenation process of propane takes place according to an oxidation-reduction mechanism (Mars–Van–Krevelen) which involves two major stages, illustrated in Fig. 9. In the first stage, propane recovers the catalyst. At this stage, the network's oxygen begins to react with propane. In the second stage, the oxygen detached from the catalyst structure is substituted by oxygen adsorbed onto the catalyst. The products, which have been adsorbed chemically, go through two different paths: they are either discharged or remain on the surface and then oxidize into other products such as CO_*x*_.^48,49^

As the catalytic test results in Fig. 8 show, propane conversion grows as the reaction temperature increases. Furthermore, propylene selectivity diminishes, followed by elevation of selectivity of CO_*x*_ (CO + CO_2_). According to the proposed reaction mechanism, propylene is produced during the oxidative dehydrogenation reaction of propane, where there is a possibility for oxidation of propane and/or propylene to CO_*x*_, as well.^10^

Calcined TNT alone showed negligible activity in the reaction and propane conversion remained almost zero in the temperature range of 200 °C to 400 °C. However, it reached propane conversions of 1.9% and 2.6% at 450 °C and 500 °C, respectively, while propylene selectivities were 85.2% and 77.3%. The activity of the nanotubes at these temperatures can be attributed to thermal cracking arising at 450 °C and above. As molybdena loading increased from 5 wt% to 10 wt%, greater conversion was observed at a constant temperature. This observation has also been reported in other literature.^25,30^ Compared to the other catalysts, MoTNT-15 showed less activity as the degree of propane conversion diminished. This can be attributed to the presence of rutile phase as well as crystalline species of molybdena. This issue has also been proven by XRD and Raman analyses. It has been reported that rutile phase has a lower capacity to maintain metal oxide species on its surface compared to anatase. Consequently, lower activity was also observed in ODH of alkanes in presence of rutile phase.^50,51^ Propylene selectivity showed a descending trend with molybdena loading increase at a constant temperature. This decrease is low for MoTNT-10 catalyst. However, MoTNT-15, for the previously mentioned reasons, displayed an obvious reduction in selectivity of propylene compared to MoTNT-10 catalyst. Propylene selectivity increased for the synthesized catalysts compared to VO_*x*_/TNT catalysts,^10^ while VO_*x*_/CeTNT showed a better selectivity towards propylene.^52^ The MoTNT-10 catalyst with propane conversion of 23.8% and selectivity of 39% has the highest propylene yield of 9.3% among the catalysts tested at 500 °C. It can be stated that an optimal presence of molybdena species is required to achieve desirable efficiency in oxidative dehydrogenation of propane in the presence of MoO_*x*_/TNT catalysts. In fact, in addition to adequate and effective presence, good dispersion of monomeric and polymeric genera should be observed, in addition to the absence of crystalline species of molybdena which cause less access to active sites of the surface.^20^

The catalytic test results of MoK0.1TNT-10 catalyst can be observed in Fig. 8. The presence of potassium caused lower conversion of propane and more selectivity for propylene compared to MoTNT-10 catalyst, which is consistent with results obtained previously.^23,29,53^ Propylene molecules may have interactions at the catalyst surface through formation of weak hydrogen bonds with surface OH groups. If these groups show Brønsted acidic property, their protons form hydrogen bonds with the π bonds of olefins. Further, when the acidic property is very powerful, proton transfer may occur from the surface to olefin, thereby forming a carbocation. This begins a set of reactions which cause reduction of selectivity.^54^ Indeed, by decreasing the acidity of the catalyst, potassium facilitates discharge of propylene (as a nucleophile) off the catalyst surface. In this way, it prevents further oxidation of intermediate products such as propylene, which causes increase in its selectivity.^53,55^ In addition, it has been suggested that the presence of potassium causes lower electrophilic oxygen concentration (O_2_^–^, O^−^). These species are highly radical and cause progressive oxidation of the products. It has also been stated that nucleophilic species of O^2−^ (as a factor of partial oxidation) increase in the presence of potassium.^53,55,56^ All the aforementioned points cause decreased catalytic activity and increased selectivity toward propylene. These results are in agreement with those observed in H_2_-TPR analysis, where the maximum reduction temperature increased in the presence of potassium. MoK0.1TNT-10 catalyst, with a propane conversion of 21.2% and propylene selectivity of 53.3%, has a yield of around 11.3% at 500 °C, which is an increase of over 20% in comparison with MoTNT-10 catalyst.

According to Fig. 8d, at a constant temperature, the largest TOF is related to MoTNT-5, the catalyst with the least molybdena loading amount, and the lowest TOF belongs to MoTNT-15 catalyst. A relationship between the maximum reduction temperature (*T*_max_) determined by H_2_-TPR and the TOF values may be established, such that the highest *T*_max_ corresponds to the sample with the lowest TOF.

### 3.8. Deactivation of catalysts

**Fig. 10:**
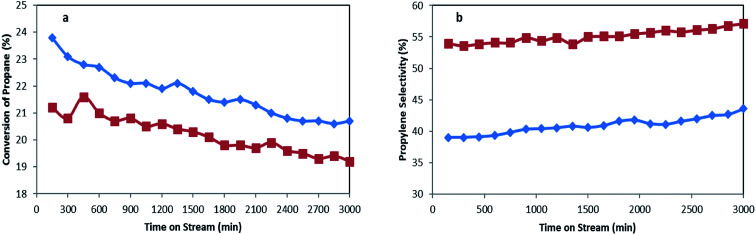
(a) Conversion of propane and (b) propylene selectivity for MoTNT-10 (♦) and MoK0.1TNT-10 (■) at 500 °C.

Performances of MoTNT-10 and MoK0.1TNT-10 catalysts, which had the greatest yield among the catalysts, were evaluated for 3000 min at 500 °C. Balance of carbon did not obviously change. Propylene selectivity and conversion of propane are provided in Fig. 10 in terms of time for both catalysts. As is shown, the catalysts had slight drops in activity. The drops in propane conversion for MoTNT-10 and MoK0.1TNT-10 are 13% and 9.1%, respectively, while both catalysts showed steady increases in propylene selectivity. This proves that the presence of potassium within the structure of molybdena increases catalyst stability.

Based on the results obtained from BET analysis provided in Table 1, the specific surface area of the MoTNT-10 catalyst is reduced to 56 m^2^ g^−1^ following 3000 min time on-stream. This reduction can be attributed to increase of the mean diameter of the crystals on the surface of the catalyst, as well as destruction of anatase phase. The specific surface area of MoK0.1TNT-10 catalyst diminished to 67 m^2^ g^−1^ after the deactivation test, showing superior stability compared to MoTNT-10 catalyst. It seems that potassium doping is absolutely effective in prevention of structural changes and reduction of surface area compared to non-doped sample. Molybdena crystal development plays the main role in specific surface area reduction of the spent catalyst, while anatase to rutile phase conversion was not observed based on the XRD pattern of spent MoK0.1TNT-10 catalyst in Fig. 2f.

## 4. Conclusion

Titanate nanotubes, synthesized by hydrothermal method, were employed as a support for molybdena and K-doped molybdena species as a catalyst in ODH of propane. Several different characterization methods confirmed the presence of titanate nanotubes and the high surface area of the support. Catalyst preparation was accompanied by support surface area loss, mainly due to calcination. The surface area reduction intensified as molybdena polymeric and crystalline species formation and support phase destruction were observed through molybdena loading up to 15% on the support. All of the tested catalysts were active, but the highest yield of propylene was obtained for the catalyst MoK0.1TNT-10, clearly different from other catalyst yields, especially at high temperatures. Addition of potassium led to much better dispersion of molybdena species on the support surface, as well as a superior yield of propylene and lower propane conversion. The catalysts showed a slight drop in propane conversion and a slighter enhancement in propylene selectivity after 3000 min on-stream, which is in agreement with specific surface area reduction through the deactivation test. MoK0.1TNT-10 catalyst showed enhanced stability in comparison to MoTNT-10 catalyst, which is attributed to better dispersion of molybdena species in the crystal structure.

## Conflicts of interest

There are no conflicts to declare.

## Supplementary Material
